# Butorphanol in Labour Analgesia

**DOI:** 10.31729/jnma.3905

**Published:** 2018-12-31

**Authors:** Jyotsna Yadav, Mohan Chandra Regmi, Pritha Basnet, KM Guddy, Balkrishna Bhattarai, Prakash Poudel

**Affiliations:** 1 Department of Obstetrics and Gyecology, B.P. Koirala Institute of Health Sciences, Dharan, Nepal; 2 Department of Anesthesiology, B.P. Koirala Institute of Health Sciences, Dharan, Nepal; 3 Department of Pediatrics, B.P. Koirala Institute of Health Sciences, Dharan, Nepal

**Keywords:** *Butorphanol*, *Labour analgesia*, *labour pain*, *numerical pain analogue score*

## Abstract

**Introduction:**

Labour is the process where uterine contractions lead to expulsion of product of conception through the vagina into the outer world. Labour pain is one of the most severe pains which has ever been evaluated and its fear is one of the reasons women wouldn't go for natural delivery. Delivery is a painful experience for all of the women except a few of them. The labor pain results from some physiological-psychological causes. Different pharmacological and non-pharmacological methods have been tried for pain relief in labour. The objective of this study is to see the effect of butorphanol injection in labour pain.

**Methods:**

It is a descriptive cross-sectional study conducted in B.P. Koirala institute of health sciences. We observed 200 pregnant women meeting the inclusion criteria and giving the informed consent who were on 1 mg butorphanol i.m. at the onset of active stage of labour every 4 hourly and on demand. Pain assessment was done by Numerical Pain analogue scale measured from 1 to 10. Fetal heart rate monitoring was done according to the hospital protocol. Caesarean section was performed for obstetrical indication. Neonatal outcome was evaluated by on duty pediatrician and APGAR score were noted at 1 and 5 min.

**Results:**

The pain scores in first, second, third, fourth hour were (8.83+0.773), (9.84+0.544), (9.94+0.338), (9.6+0.298) respectively, where 1^st^ and 2^nd^ hour is statistically significant.

**Conclusions:**

Butorphanol is an effective labour analgesia without significant adverse effects on women and the neonatal outcome.

## INTRODUCTION

Labor pain is one of the most severe pains and its fear is one of the reasons women wouldn't go for natural delivery. Different analgesics have been used like opioids, inhaled analgesia, parenteral opioids have been used. Butorphanol tartrate is a synthetically derived opioid agonist-antagonist analgesic of the phenanthrene series. It is a new synthetic opioid which has a partial agonistic action, has emerged as a promising agent in terms of efficacy and better safety profile.

Butorphanol intramuscular injection can be a good possible option for pain relief in labour with minimum monitoring with almost no effect on neonatal outcome and can be used in low resource setting with less expertise. It is one easy and cheap method and without need of invasive procedures.^1^

The objective is to study the effect of butorphanol in labour analgesia. Primary Outcome is to rate pain relief following intramuscular injections of butorphanol. Whereas secondary outcome is to see need of additional analgesia, duration of labour, mode of delivery and APGAR score at 1 and 5 min, NICU Admissions.

## METHODS

It is a descriptive cross-sectional study conducted in the Department of Obstetrics and Gynecology at B.P. Koirala Institute of Health Sciences, Dharan, Nepal over 12 months duration (1^st^ September 2015 to 31^st^ August 2016). Ethical clearance was obtained from Institutional Review Committee (IRC-BPKIHS) before starting this study. Verbal and written consents were obtained from the participants who were enrolled in the study. All pregnant women admitted to BPKIHS labour ward following spontaneous onset of labour meeting the inclusion criteria were included in the study.

### Inclusion criteria

- Pregnant women at term period of gestation (at 37 to 42 weeks period of gestation)
- Pregnant women in active stage of labour following spontaneous onset of labour.

### Exclusion criteria

- All pregnant women having medical risk factors like Hypertension, Asthma, Diabetes, Obesity (BMI = >30), psychiatric illness, cardiac disease.
- Obstetric complications: Breech, multiple pregnancy, small for gestation fetus, previous uterine surgery, previous caesarean section.
- Participants with meconium stained liquor.
- Participants on epidural anesthesia.
- Preterm labour.
- Hypersensitivity to opioids.
- Any contraindication for normal vaginal delivery.

Sample size was calculated by using the formula given below


$$\begin{array}{l} \text{Sample size }(\text{n})={\text{z}}^{2}\times \text{p}\times \text{q}/{\text{d}}^{2}\\ =1.62\times 1.62\times 0.5\times (1-0.5)/{\left(10/100\right)}^{2}\\ =1.62\times 1.62\times 0.5\times 0.5/{\left(0.1\right)}^{2}\\ =0.6561/0.001\\ =65.61 \end{array}$$

Where,

z= Value of z at confidence interval of 90%
p= prevalence of study, i.e 50%
q= 1-p

And e= margin of error, 10%

This study could be biased. There could be subjective, observational and interview bias. Confounding Factor can be psychological impact on the patients and multiparity.

Enrolled pregnant women meeting the inclusion criteria and giving the informed consent were on 1 mg butorphanol i.m. at the onset of active stage of labour every 4 hourly and on demand. Pain assessment was done by Numerical Pain analogue scale (NPAS) measured from 1 to 10. The pain was rated as 10 before intervention for all the participants enrolled in the study. After interventions women were verbally asked to rate their reduction in labour pain from 1 to 10 every hourly till delivery. Artificial rupture of membrane was done for all women in active stage of labour. Fetal heart rate monitoring was done. Caesarean section was performed for obstetrical indication. Women were monitored for one hour in labour room after delivery. Need of additional analgesia, time of delivery, mode of delivery, and duration of delivery was also noted. Neonatal outcome was evaluated by on duty pediatrician and APGAR score were noted at 1 and 5 min. The conditions of baby and mother were followed till discharge.

Relevant data were entered from proforma in Microsoft Excel 2013 and a master chart was prepared. All the data were exported to the software SPSS version 11.5 and descriptive statistical analysis was done.

## RESULTS

Total of 200 parturient women with more than 37 weeks of gestation, in active stage of labour were enrolled in the study. All participants completed the study. All pregnant women aged above 16 years participated it the study. The women who received injection Butorphanol ranged from 37 weeks to 42 weeks of gestation including both primigravida and multigravida.

The women received injection butorphanol at cervical dilatation of 4 cm and above.

**Table 1:** Showing cervical dilatation of the women.

Characteristics	Category	n (%)
Dilatation (in cm)	4–6	154 (77)
	7–10	46 (23)
Total		200

Pain reduction was seen in 99% of subjects. Pain scoring was done using ten points Numerical Pain Analogue Scale (NPAS) before, and every hour after intramuscular injection of 1mg butorphanol in active stage of labour. Significant pain reduction was observed after 1 hour and 2 hour of injection. However, after 3 and 4 hour of injection, the pain reduction was not seen to be significant. This could be because the peak analgesic effect of intramuscular administration of butorphanol is seen within 30 to 60 minutes.

**Table 2:** Showing Numerical Pain Analogue Scale at different interval.

	n	Minimum	Maximum	Mean	Std. Deviation
NPAS before*	200	10	10	10.00	0.000
NPAS 1^†^	200	5	10	8.83	0.773
NPAS 2^‡^	165	7	10	9.84	0.544
NPAS 3^§^	77	8	10	9.94	0.338
NPAS 4^∥^	45	8	10	9.96	0.298

NPAS before^*^:- Numerical pain analogue scale before injection

*NPAS 1^†^:- Numerical pain analogue scale after one hour of injection*

*NPAS 2^‡^:- Numerical pain analogue scale after two hours of injection*

*NPAS 3^§^:- Numerical pain analogue scale after three hours of injection*

*NPAS 4^∥^:- Numerical pain analogue scale after four hours of injection*

Most of the participants delivered vaginally between two to four hours of injection of butorphanol as shown in the graph. Whereas four patients underwent caesarean section. Out of which two patients had LSCS between three to four hours and other two after five hours of injection butorphanol.

**Figure 1.**
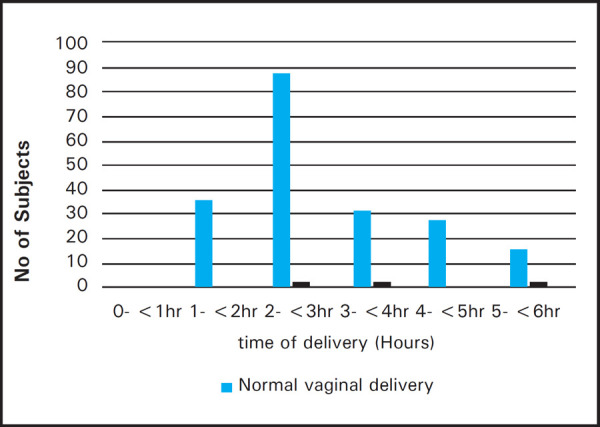
Showing time of deliveries.

Among 200 participants, 4 had undergone lower segment cesarean section while rest had normal vaginal delivery.

**Table 3:** Showing different modes of delivery.

		n (%)
Mode of Delivery	Vaginal	196 (98)
	LSCS	4 (2)

Four of the participants had undergone LSCS. The indication for LSCS was meconium stained liquor in two of them, while it was arrest of descent and dilatation in the other two.

**Table 4:** Showing the indications for LSCS.

	Indication	n (%)
Indication for LSCS	Meconium stained liquor	2 (50)
(n = 4)	Arrest of descent and dilatation	2 (50)

The neonatal outcome of all the women who received injection butorphanol is shown below:

**Table 5:** Showing newborn characteristics among delivered.

n (%)		
Neonatal outcome	Alive	200 (100)
APGAR score	Normal	200 (100)
NICU admission	No	199 (99.5)
	Yes	1^*^(0.5)

* *The neonate was admitted to NICU for perinatal depression.*

## DISCUSSION

There are various pharmacological and non-pharmacological methods of pain relief in labour. This study was conducted to see the effectiveness, feasibility, and simple as well as with fewer side effects to the fetus so that the labouring patients can have a good pain relief during delivery.

Opioids commonly used for labour analgesia are meperidine, morphine, fentanyl, nalbuphine and butorphanol. All of these are potent analgesics, but fear of the above side effects may restrict their widespread use.

Butorphanol tartrate is a potent analgesic with partial agonist action with minimum side effects. Butorphanol tartrate is a synthetically derived opioid agonist-antagonist analgesic of the phenanthrene series. Butorphanol is a mixed agonist-antagonist with low intrinsic activity at receptors of the //-opioid type (morphine-like). It is also an agonist at k-opioid receptors.

Several studies have been conducted to see the analgesic effect of butorphanol in labour pain.

The present study demonstrated the analgesic effects of butorphanol. The study is a descriptive cross-sectional study and was conducted in a sample of 200, which is a large sample size. The study included pregnant women with gestational age more than 37 weeks to high as 42 weeks and conducted above 16 years onwards. Two hundred subjects were given 1 mg injection butorphanol in active stage of labour and pain relief was noted on a ten point numerical pain analogue scale (NPAS) which showed reduction in pain score in first hour of administration of 1 mg of intramuscular injection of butorphanol. APGAR scores were noted at 1 and 5 minutes and showed that there was no effects on the neonatal outcome. Some of the women had sedation for long time but did not require antidote or respiratory support. There was no incidence of drug hypersensitivity or respiratory depression.

Similar study was conducted by Halder A et al^2^ in which one hundred low risk term consenting pregnant women were recruited to take part in a prospective cohort study. Intramuscular injections of butorphanol tartrate 1 mg were given in the active phase of labour and repeated two hourly. Pain relief was noted on a 10-point visual pain analogue scale (VPAS). Obstetric and neonatal outcome measures were mode of delivery, duration of labour, Apgar scores at 1 and 5 minutes and Neonatal Intensive Care Unit admissions and concluded that butorphanol is an effective parenteral opioid analgesic which can be administered with reasonable safety for the mother and the neonate. The study has the drawback of lack of control and small sample size where as in this study a large sample size was taken. Similarly the study included pregnant women above 37 weeks of gestation. Majority of the women who participated in the present study were between 25 to 30 years where as in this majority of the women were between 19 to 25 years of age. About 98% achieved normal vaginal delivery and four participants had cesarean section whereas in the study performed by Haldar et al 90% delivered vaginally and two had cesarean section. Seventeen percent of women received one dose and 21.5% received two doses, whereas in the study done by Haldar et al 86% received two doses, 13% were given three doses and one received only one dose. There was significant decrease in pain after 15 minutes of administration and remained low for six hours whereas this study showed significant decrease in pain after one and two hours of administration.

This study showed that there was 99% reduction in labour pain in women. Similarly Nelson Kennethe E et al^3^ studied the effect of intravenous butorphanol and meperidine and their combination in labouring women in a randomized control trial and concluded that all three treatments reduced pain intensity equally. Twenty-nine of women exhibited clinically meaningful pain relief, with no difference among groups. Butorphanol, meperidine, and their combination reduced pain intensity similarly by an average of 25–35%. During first stage of labour, many women suffer from lower back pain. Intramuscular injection butorphanol had significant effect in pain reduction in first and second hour of injection. Similar study done by Kirti and Saxena et al^4^ in which patients received 4 intracutaneous injections of sterile water or normal saline 0.5ml in the lumbo-sacral region. Pain scores, progress of labour and fetal outcome were studied. There was significant reduction of pain scores in the sterile water group but not in the normal saline group at 10, 45 and 90 minutes after injection. There was no difference in the progress of labour and fetal outcome between the two groups. Likewise this study also showed no delay in time of delivery and no effect on neonatal outcome after butorphanol injection. The study had smaller sample size of 100 participants whereas this study include a large number of sample of 200 of participants. The mean age was 24.7 years, including both primigravida and multigravida with gestational age more than 37 years and the subcutaneous injection was given at 3 centimeter of cervical dilatation. Similarly the mean age in the present study is 24.8 years with gestational age of the women were more than 37 years and injection butorphanol was injected in active stage of labour after 4 centimeter of cervical dilatation. Similarly MYK Wee et al^5^ conducted a two centered randomized blinded control trial in which intramuscular (i.m.) pethidine used for labour analgesia and i.m. diamorphine used in labouring women. This trial aimed to ascertain the relative efficacy and adverse effects of diamorphine and pethidine for labour pain. Diamorphine provided modestly improved pain relief at 60 minutes which was noted on 10-cm visual pain analogue scale. Likewise present study showed reduction in pain relief within 60 minutes of i.m. butorphanol and pain relief was noted on 10 point numerical analogue scale. The women were included above 37 weeks of gestation to as high as 42 weeks with singleton pregnancy which was similar to the present study. Hutton et al^6^ conducted a systematic reviews and meta analysis of randomized control trials on sterile water injection for labour pain included 8 randomized control trials. This study studied the effect of sterile water on low back pain and decrease or increase in caesarean section rate. The Caesarean section rate was 4.6% in the sterile water injection group and 9.9% in the comparison group (n = 828)(RR 0.51, 95% CI:0.30, 0.87) and found that there was significant decrease in the rate of Caesarean section when sterile water injections were used to manage low back pain of labour. The pain was measured using visual pain analogue scale. Similarly in the present study there were only four caesarean section.

Limitations of the study could be small sample size and also effect of butorphanol on mode of delivery could have been other good outcome evaluation of which was not done.

## CONCLUSIONS

This study provides evidence that administration of intramuscular injection of butorphanol is effective in reducing labour pain without significant side effects to the women. Most of the women delivered within six hours of injection butorphanol, with almost no effects on neonatal outcome with good APGAR score at birth. One NICU admission was required for perinatal depression. Mode of delivery in 98% of the women was normal vaginal delivery. Women were much satisfied with the procedure who were enrolled in the study. Therefore intramuscular injection of butorphanol can be used as an easy and feasible method of labour analgesia in women.

## Conflict of Interest

**None.**
